# Common laboratory diets differentially influence zebrafish gut microbiome’s successional development and sensitivity to pathogen exposure

**DOI:** 10.21203/rs.3.rs-2530939/v1

**Published:** 2023-02-02

**Authors:** Michael Sieler, Colleen Al-Samarrie, Kristin Kasschau, Zoltan Varga, Michael Kent, Thomas Sharpton

**Affiliations:** Oregon State University; Oregon State University; Oregon State University; University of Oregon; Oregon State University; Oregon State University

**Keywords:** Zebrafish, Gut microbiome, Development, Infection, Diet, Husbandry, Mycobacterium chelonae

## Abstract

**Background:**

Despite the long-established importance of zebrafish *(Danio rerio*) as a model organism and their increasing use in microbiome-targeted studies, relatively little is known about how husbandry practices involving diet impact the zebrafish gut microbiome. Given the microbiome’s important role in mediating host physiology and the potential for diet to drive variation in microbiome composition, we sought to clarify how three different dietary formulations that are commonly used in zebrafish facilities impact the gut microbiome. We compared the composition of gut microbiomes in approximately 60 AB line adult (4- and 7-month-old) zebrafish fed each diet throughout their lifespan.

**Results:**

Our analysis finds that diet has a substantial impact on the composition of the gut microbiome in adult fish, and that diet also impacts the developmental variation in the gut microbiome. We further evaluated whether the 7-month-old fish microbiome compositions that result from dietary variation are differentially sensitive to infection by a common laboratory pathogen, *Mycobacterium chelonae*. Our analysis finds that the gut microbiome’s sensitivity to *M. chelonae* infection varies as a function of diet, especially for moderate and low abundance taxa.

**Conclusions:**

Overall, our results indicate that diet drives the successional development of the gut microbiome as well as its sensitivity to exogenous exposure. Consequently, investigators should carefully consider the role of diet in their microbiome zebrafish investigations, especially when integrating results across studies that vary by diet.

## Introduction

In the effort to understand how the gut microbiome mediates vertebrate health, zebrafish *(Danio rerio*) have emerged as an important microbiome experimental model organism[1]. Despite the increasing use of zebrafish in microbiome research, key knowledge gaps remain about how different zebrafish husbandry practices, especially diet, influences microbiome composition[2, 3]. For example, in contrast to mice, zebrafish do not have a standard reference diet[4]. Instead, zebrafish research facilities vary by dietary husbandry practice, which can impact physiological and reproductive outcomes[5–7]. Given that diet plays an important role in shaping the composition of the gut microbiome in humans and across vertebrate and invertebrate animal models, such as mice and honeybees[8–13], we hypothesize that variation in dietary husbandry practice also impacts the composition of the zebrafish gut microbiome. Quantifying this association is important because it could explain why, despite the existence of a core gut microbiome, gut microbiome composition differs across research facilities[14, 15], improve efforts to integrate data across investigations, and clarify how dietary variation manifests as physiological variation.

Relatively little is known about how variation in dietary husbandry practice impacts the zebrafish gut microbiome. Prior studies that measured the impact of diet on the zebrafish gut microbiome have largely considered how substantial variation in specific macronutrients impacts the gut microbiome (e.g., high fat versus low fat diets)[6, 16–18]. This variation is not typically representative of the variation in nutrient content observed across standard dietary husbandry practices[4, 5]. Additionally, these studies have typically reared fish on a singular diet up to the point of experimentation, at which point fish are exposed to alternative diets. While insightful about acute effects, such experimental designs do not model the chronic dietary exposure that fish experience through husbandry. This prior work also does not typically consider how diet impacts the microbiome at different fish developmental periods, or whether dietary variation affects other characteristics of the gut microbiome, such as its sensitivity to exogenous agents (e.g., pathogens).

In this study, we sought to define the impact of rearing fish of different common facility diets on the gut microbiome of early adult (4-mo old) and fully mature (7-mo old) zebrafish. To do so, we reared fish throughout their lifespan on one of three different dietary husbandry practices: fish were fed either (1) the Gemma (Skretting, Fontaineles-Vervins, France) diet, which is a commercial feed widely used in zebrafish research facilities, (2) the ZIRC diet, a compound diet mixed and adopted by the Zebrafish International Research Center (ZIRC), which is one of the largest zebrafish stock centers in the world, or (3) a precisely defined laboratory grade diet developed by Watts[5]. Overall, these diets are relatively similar from a macronutrient perspective, though they differ by formulation, ingredient sourcing, manufacturing process details, and consequently also by exact nutritional content. (Table S4.1.1). In particular, we evaluated how the microbiome differed across these groups of fish as well as over development. We also determined if these differences link to variation in fish weight and condition factor, as well as variation in how the microbiome responds to infection by one of the most common infectious agents of zebrafish research facilities, *Mycobacterium chelonae*[9].

## Results

### 1. Diet differentially influences physiology and gut microbiome at 4 months old

**Figure 1:**
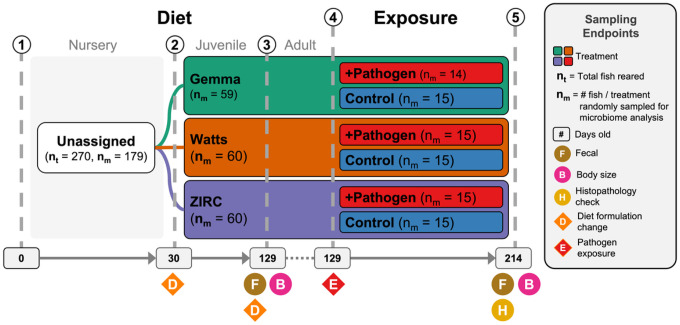
Experimental design showing treatments and husbandry events during the course of the study. Symbols indicate when an event occurred. 1) 270 fish were reared from 0 to 30 days post fertilization (dpf) on a nursery diet across 18 tanks (15 fish per tank). 2) At 30 dpf, fish were assigned one of three diets (e.g., Gemma, Watts, or ZIRC), and fed a juvenile formulation until 129 dpf. 3) At 129 dpf, fish were switched to an adult formulation of their respective diets. Additionally, body size measurements were conducted on all fish and fecal samples were collected from a random selection of five fish per tank (n = 90). 4) Afterwards, a cohort of fish from each diet were exposed to *Mycobacterium chelonae*. 5) Three months later when fish were 214 dpf, body size measurements were conducted on all fish and fecal samples were collected from a random selection of five fish per tank (n = 89). Histopathology check was conducted to assess infection burden on all fish.

To determine how common zebrafish diets differently impact fish size (length and body condition score) and the gut microbiome, we reared 176 zebrafish that were assigned one of three diets from 1- to 4-months-post fertilization (mpf) (Figure 1): Gemma, Watts and ZIRC diets. Prior to diet assignment, fish were fed a nursery diet (see methods). At 4 mpf, we selected 89 individuals across these three cohorts and collected fecal samples from each fish for microbiome profiling prior to measuring their weight and body condition score (BCS). Wilcoxon Signed-Rank Tests found that diet and sex significantly associated with weight and BCS. Female fish had higher weight (Z = 1,530, P < 0.001; Table S1.1.2) and BCS (Z = 1,631, P < 0.001; Table S1.1.4) compared to males. Between the three diets, ZIRC-diet fed fish had the highest mean BCS compared to fish fed Gemma- (Z = 150, P < 0.001) and Watts-diet (Z = 197, P < 0.001, Table S1.1.3). Gemma- and Watts-diet fed fish did not significantly differ from one another in terms of weight and BCS. These results indicate that ZIRC-diet contributes to heavier fish compared to Gemma- and Watts-diet fed fish.

We next built generalized linear models (GLM) to determine if diet associated with variation in one of three measures of microbiome alpha-diversity: richness, Simpson’s Index, and Shannon Entropy. An ANOVA test of these GLMs revealed that alpha-diversity varies as a function of diet for all three measures of diversity we assessed (P < 0.05; Fig 1C; Table S1.2.1). A post hoc Tukey test clarified that ZIRC- and Watts-diet fed fish exhibited significant differences in alpha-diversity as measured by richness and Shannon Entropy (P < 0.001, Table S1.2.2). Moreover, we observed significant differences in diversity between Gemma- and Watts-diet fed fish in terms of richness (P < 0.001; Table S1.2.2), and between Gemma- and ZIRC-diet fed fish when considering the Simpson’s Index (P < 0.001; Table S1.2.2). These results indicate that diet associates with fish gut microbiome diversity, and that diet may differentially impact rare and abundant microbial members of the gut.

**Figure 2:**
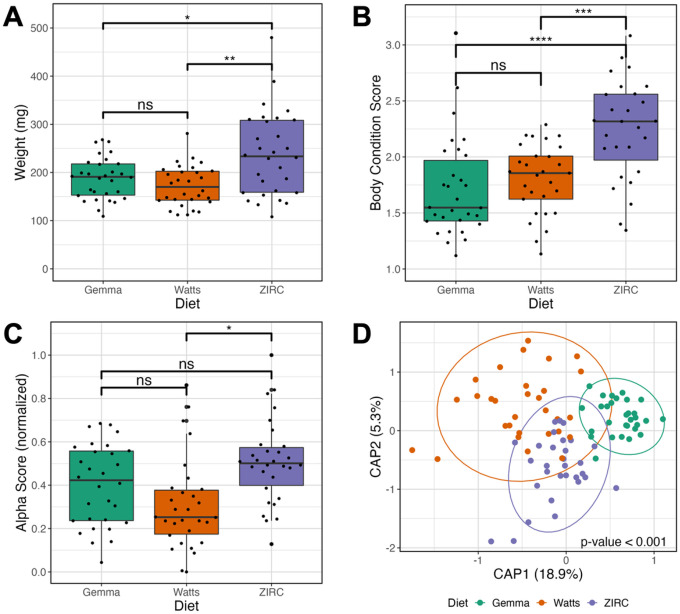
Effects of 4 months post fertilization (mpf) fish fed one of three diets (Gemma, Watts, or ZIRC) on physiology and microbiomes of zebrafish. (**A**) Weight of ZIRC-diet fed fish significantly differs from Watts- and Gemma-diet fed fish. Gemma- and Watts-diet fed fish do not differ from each other. (**B**) Body condition score is a length normalized measure of weight. Fish fed the ZIRC diet have significantly higher body condition scores from fish. fed the Gemma and Watts diets. (**C**) Shannon Entropy of diversity shows that gut microbiome diversity significantly differs between Gemma- and Watts-diet fed fish, ZIRC- and Watts-diet fed fish, but not between Gemma- and ZIRC-diet fed fish. (**D**) Capscale ordination based on the Bray-Curtis dissimilarity of gut microbiome composition. The analysis shows that physiology and gut microbiome composition significantly differs between the diets. “ns” indicates not significantly different, *, **, *** indicates significant differences below the 0.05, 0.01, and 0.001 levels, respectively.

To evaluate how diet associates with microbiome community composition, we quantified the Bray-Curtis, Canberra and Sørensen dissimilarity amongst all sample. We detected a significant clustering of microbial gut community composition based on diet as measured by all beta-diversity metrics (PERMANOVA, P < 0.05; Figure 2C, Table S1.3.1). These results indicate that microbial communities of fish fed the same diet are more consistent in composition to one another than to fish fed other diets. Additionally, we assessed beta-dispersion, a measure of variance, in the gut microbiome community compositions for each diet group. We find the beta-dispersion levels were significantly different between the diet groups as measured by Bray-Curtis and Canberra metrics (P < 0.05; Table S1.4.1). Beta-dispersion levels were significantly reduced in Gemma-diet fed fish compared to Watts-diet fed fish when measured by Bray-Curtis metric, as well as significantly reduced compared to Watts- and ZIRC-diet fed fish when measured by Canberra metric (Table S1.4.1). These results indicate that Gemma-diet fed fish are more consistent in community composition than Watts- and ZIRC-diet fed fish at 4 mpf. Collectively, these results indicate that 4 mpf fish gut microbiome communities stratify by diet, but the composition of these microbial communities differ in consistency depending on diet.

Finally, to better understand the interactions between the diet and the members of the gut microbiome community, we quantified differential abundance using ANCOM-BC2. We observed 24 significantly abundant taxa at the genus level in at least one of the three diets (Table S1.5.1). Gemma-diet fed fish were enriched for *Chitinibacter* and were depleted of *Aeromonas* and *Fiavobacterium*. Watts-diet fed fish enriched for *Fiavobacterium, Z0R0006, Peptostreptococcus, Cetobacterium, Tabrizicola, Cellvibrio*, and unnamed genera of *Microscillaceae* and *Chitinibacteraceae*, and depleted of *Crenobacter* and a *Sutterellaceae* genus. ZIRC-diet fed fish enriched for *Cloacibacterium* and *Acinetobacter*, and depleted of *Fluviicola*. Many of these taxa are identified as common members of the zebrafish gut microbiome[14, 15]. These results indicate that diet differentially supports particular members of the zebrafish microbiome community.

### 2. Diet impacts the successional development of the zebrafish gut microbiome

**Figure 3:**
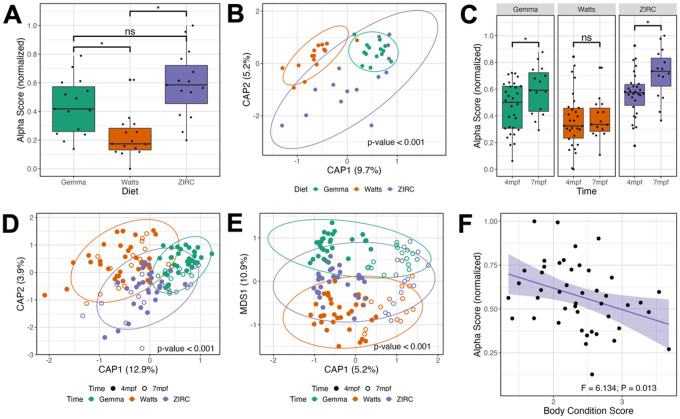
Development is associated with altered microbiome composition. (**A**) Shannon Entropy of diversity shows that gut microbiome diversity significantly differs between Watts-diet fed fish to fish fed the Gemma- and ZIRC-diets in 7 months post fertilization (mpf) zebrafish. (**B**) Capscale ordination based on the Bray-Curtis dissimilarity of gut microbiome composition in 7 mpf zebrafish. (**C**) Shannon Entropy for diversity shows microbial gut diversity increases with development in 4 to 7 mpf zebrafish fed the Gemma- and ZIRC-diets, but not Watts-diet fed fish. Capscale ordination of gut microbiome composition based on the (**D**) Bray-Curtis dissimilarity by diet and (**E**) Canberra measure by time. (**F**) Body condition score negatively associates with gut microbiome diversity as measured by Simpson’s Index across 4 and 7 mpf zebrafish fed. the ZIRC diet. The analysis shows that fish size and gut microbiome composition significantly differs between the diets across development, and there may be diet-dependent link with physiology. A “ns” indicates not significantly different, “*” indicates significant differences below the 0.05 level.

To determine how maintaining fish on different diets impacts the development of the gut microbiome, we continued to grow fish from the same diet cohorts until 7 months post fertilization (mpf; Figure 1). Microbiome samples were collected from cohort members prior to quantification of fish weight and body condition score. To determine the effect of diet on the body condition score and the gut microbiome of 7mpf fish, we conducted the same analyses as we applied to the 4 mpf fish. At 7 mpf, we find body condition score is significantly associated with diet (P < 0.05; Table S2.1.3.1). Additionally, linear regression analyses revealed statistically significant main effects of diet on gut microbiome alpha- and beta-diversity for all metrics we considered (P < 0.05; Fig 3A&B, Table S2.1.3.2–3), but an ANOVA test of beta dispersion was not significantly different between diets for any beta-diversity metric (P > 0.05; Table S2.1.3.4). These results demonstrate that diet impacts the physiology and gut microbiome of 7mpf fish.

Next, we compared our results between the 4 and 7 mpf fish to determine how diet impacts the successional development of the gut microbiome. Linear regression revealed microbial gut alpha-diversity was significantly associated with the main effect of time (P < 0.05; Table S2.2.1) for each diversity metric. However, we did not find a diet dependent effect on time for any alpha-diversity metric we assessed (P > 0.05; Table S2.2.1). A post hoc Tukey test clarified that microbiome diversity was significantly different between 4 and 7 mpf Gemma- and ZIRC-diet fed fish as measured by the Shannon and Simpson’s alpha-diversity metrics (P < 0.05; Figure 3C, Table S2.2.2), but we did not find a statistically significant association between 4 and 7 mpf Watts-diet fed fish with any alpha-diversity metric (P > 0.05; Table S2.2.2). These results indicate that the alpha-diversity of the gut microbiome of Watts-diet fed fish were temporally stable, while Gemma- and ZIRC-diet fed fish diversified over time in diet-consistent ways.

A PERMANOVA test of the 4- and 7-mpf samples using the Bray-Curtis dissimilarity metric revealed that community composition was best explained by diet (P < 0.05; Figure 2C, Table S2.3.1), but an analysis using the Canberra measure found that variation in microbiome composition was best explained by time (P < 0.05; Fig 2D, Table S2.3.2). Given how these metrics weight the importance of abundant versus rarer taxa, respectively, these results indicate that abundant members of the microbiome community are more sensitive to the effects of diet, while rarer community members are sensitive to the effects of time. Moreover, we found beta-dispersion levels were significantly elevated between 4 and 7 mpf Gemma-diet fish when considering the Bray-Curtis and Sørensen metrics, in Watts-diet fed fish when considering the Canberra and Sørensen metrics, and in ZIRC-diet fed fish across all three beta-diversity metrics (P < 0.05; Table S2.4.1–3). These results indicate that abundant and rarer gut microbiome community members were differentially impacted by the effects of time depending on diet. Collectively, these results indicate that diet can have a substantial impact on how the gut microbiome successionally develops in zebrafish.

Differential abundance analysis revealed taxa that were significantly associated with the effects of time and diet one of the diets (Table S2.5.1). Across all three diets, the taxa that were more abundant included *Fluviicola, Macellibacteroides, Bacteroides* and an unnamed genus in the *Barnesiellaceae family* were, while taxa that were less abundant included *Phreatobacter* and Flavobacterium. These results indicate that irrespective of diet, the abundances of taxa change over the course of zebrafish development. We also measured how taxon abundance changed over time within each diet (Figure S2.5.2–46.2.5). The Gemma-diet fed fish were uniquely enriched for *Exiguobacterium* (Table S2.5.2). *Exiguobacterium* are gram-positive facultative anaerobes in the phylum Bacillota, and are linked to fatty acid metabolism in zebrafish [20, 21]. The Watts-diet fed fish were uniquely depleted of *Gemmobacter*(Table S2.5.3). Previous work has found that *Gemmobacter* has a positive association with parasite exposure in infected zebrafish[22, 23]. The ZIRC-diet fed fish were uniquely enriched for *Pseudomonas* and *Haliscomenobacter*(Table S2.5.4). *Pseudomonas* is a common member of the gut microbiome and associated with fatty acid metabolism in zebrafish[20]. Less is known about the *Haliscomenobacter* genus, but an analysis of its genome revealed it is an aerobic chemoorganotroph found in aquatic systems [24]. Together, these results indicate that particular members of the gut microbiome associate with diet and zebrafish development.

To determine if fish size associated with diet across zebrafish development, we used Wilcoxon Signed-Ranks Tests to identify parameters that best explained the variation in body condition score (BCS) between 4- and 7-mpf fish. At 7mpf, the BCS significantly differed between fish fed different diets. However, we did not find that BCS of fish were impacted by time (P > 0.05; Fig 2E, Table S2.1.1). These results indicate that while fish differ in BCS between diets at 7 mpf, their weight and length grow proportionally at a similar rate from 4 to 7 mpf. Interestingly, we observed a significant negative association of BCS and microbial gut diversity uniquely in fish fed the ZIRC diet as measured by Shannon Entropy and Simpson’s Index (P < 0.05; Fig 2F, Table S2.1.2.1). This result indicates that fish gut microbiomes with higher body masses are lower in diversity compared to fish with lower body mass. For Canberra and Sørensen beta-diversity metrics, there were significant main effects of body condition score, and significant interaction effects between BCS and diet (P < 0.05; Table S2.1.2.2). However, the model coefficient for the effect of body condition score and its interaction with diet is far smaller than the coefficient for the effect of diet (Table S2.1.2.2). We did not find a significant association between BCS and specific taxon abundance (Table S2.1.2.2). Collectively, these results indicate that while the gut microbiome’s composition associates with BCS, the effect of diet on the gut microbiome is much stronger.

### 3. Diet influences gut microbiome’s sensitivity to pathogen exposure

Lastly, we sought to determine how diet impacts the gut microbiome’s sensitivity to exogenous stressors, in particular exposure to the common pathogen of zebrafish, *Mycobacterium chelonae*. Mycobacteria has been reported in zebrafish from about 40% of research facilities[25].

The infection is usually only diagnosed by histology, and hence s only diagnosed to the genus level based on the presence of acid-fast bacteria. When species identifications are made using molecular methods, the identification is most frequently M. chelonae[26]. It is hypothesized to be introduced through diet early in life[25, 27, 28]. *M. chelonae forms* granulomas coelomic organs, swim bladder and kidney, and in many cases it ultimately causes death. These can introduce inconsistencies in study outcomes, but the impacts on the gut microbiome are not known[25]. To clarify effect of *M. chelonae* infection on the gut microbiome, and whether these effects vary by diet, we injected *M. chelonae* into the coelomic cavities of fish from each diet cohort at 4 mpf following fecal collection. These *M. chelonae* injected fish comprised the pathogen exposure cohort for this experiment, which we compared to the remaining, unexposed cohort of fish. At 7 mpf, we collected fecal samples from exposed and unexposed fish to measure microbial gut diversity, composition, and taxon abundance, performed a histopathological analysis of intestinal tissue to assess infection severity, and measured body condition score.

We first evaluated whether diet impacted infection outcomes, as determined by histological confirmation of infection 3.5 months following pathogen injection. We conducted a Chi-Square test to compare the infection count between fish fed the three diets. The results showed that there was a statistically significant difference in proportion of positive infection counts between the groups, X^2^ (2, N = 66) = 11.519, P < 0.05 (Table S3.1.1). Across all three diets, all females had infected ovaries. In contrast, we observed the following infection outcomes for male fish with extra-intestinal infections Gemma 3/12 (25%), Watts 5/24 (20.8%), and ZIRC 18/24 (70.6%) (Table 3.5.2.2.1). In male fish only, we also found a statistically significant difference in proportion of infected fish across the three diets (X^2^ = 11.556, df = 2, N = 53, P < 0.05; Table S3.1.2). When we conduct the same analysis with just fish sampled for microbiome analysis (Table S3.5.1.3.1), we do not observe significant effects (X^2^ = 4.069, df = 2, N = 44, P > 0.05; Table S3.1.3), likely due to being underpowered to detect these effects. Infections in males included the testis, coelomic cavity, swim bladder and kidney (Figure S3.1.2). With females, all showed the infections within the ovaries, with one with a coelomic infection. Colonization of the intestinal lumen by acid fast bacteria were observed in 17 exposed and 7 control fish across the diets. This result indicates that the diets considered in our study appear to dictate the progression of infection of *M chelonae*, but of the samples we collected for microbiome analysis we may be underpowered to detect a difference. Next, we assessed whether infection status links to body condition score as well as measures of gut microbiome diversity and composition. We did not observe significant associations between infection status and body condition score based on linear regression (P > 0.05; Table S3.1.4) or any of the gut microbiome diversity and composition measures (P > 0.05; Table S3.1.5 & S3.1.6). Together, these results indicate that infection endpoints are linked to diet, but not body condition score or the gut microbiome.

**Figure 4:**
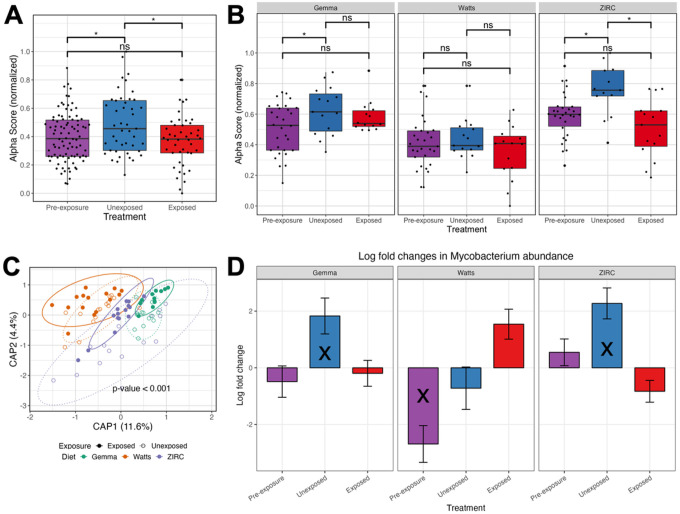
Exposure to *Mycobacterium chelonae* inhibits diversification of gut microbiome. (A) Shannon Index for diversity of pre-exposed 4 month post fertilization (mpf), 7 mpf exposed and unexposed fish, and (B) for exposure groups within each diet. Capscale ordination based on the Bray-Curtis dissimilarity of gut microbiome composition of fish by (C) diet. (D) Log fold change of *Mycobacterium* of pre-exposed, exposed and unexposed fish within each diet as calculated by ANCOM-BC. Values are in reference to exposed fish within each diet. The analysis shows gut microbiome’s sensitivity to pathogen exposure is linked to diet, but *Mycobacterium’s* abundance is diet-dependent. A “ns” indicates not significantly different, and * indicates significant differences below the 0.05. An “X” indicates a group is significantly differentially abundant compared to the exposed treatment reference group.

We next considered that exposure to the pathogen could impact the gut microbiome, even though ultimate infection outcomes among exposed individuals may not. Comparing exposed to unexposed fish found that microbial gut diversity significantly differs between exposure groups as measured by richness and Shannon Entropy alpha-diversity metrics (P < 0.05; Figure 4A, Table S3.2.1). That said, based on linear regression, the impact of exposure on the gut microbiome alpha-diversity does not appear to differ as a function of diet, as the interaction term for these covariates did not yield a significant effect (P > 0.05; Table S3.2.1). Furthermore, we used a post hoc Tukey test to clarify whether microbial gut diversity of fish differed between exposure groups by diet. Unique to ZIRC-diet fed fish, we observed microbiome diversity differed in unexposed controls compared to exposed fish as measured by all alpha-diversity metrics (P < 0.05, Table S3.2.2). Watts-diet fed fish differed in unexposed controls compared to exposed fish in terms of richness (P < 0.05, Table S3.2.2). These results suggest that the gut microbiome diversity of ZIRC-diet fed fish, and to some extent Watts-diet fed fish, are sensitive to the effects of *M chelonae* exposure, but Gemma-diet fed fish are resistant to pathogen exposure. While the gut microbiomes are sensitive to the effects of pathogen exposure, we find the statistical effect of diet shaping the gut microbiome is an order of magnitude greater across all alpha-diversity metrics (P < 0.05, Table S3.2.1). Collectively, these results indicate that gut microbiome diversity is sensitive to *Ml. chelonae* exposure, but diet is the primary driver of gut microbiome diversity.

Next, we evaluated how pathogen exposure influenced microbial community composition across fish fed each diet. For each beta-diversity metric considered, PERMANOVA tests found that the main effects of diet and pathogen exposure significantly explained the variation in microbiome composition, but that the main effect of diet was consistently larger than the effect of exposure (P < 0.05; Fig 4C, Table S3.3.1). Furthermore, a PERMANOVA test found that the model coefficient effect for the interaction of diet and pathogen exposure was statistically significant when considering Canberra and Sørensen beta-diversity metrics, however this effect was marginal as compared to the aforementioned main effects. Moreover, a pairwise analysis of beta-dispersion did not find significant levels of dispersion between exposed and unexposed fish within each diet (P > 0.05; Table S3.4.1 −3). These results indicate that exposure to *M. chelonae did* not affect dispersion of the gut microbiome communities. Collectively, these results indicate that the gut microbiome is sensitive to pathogen exposure, but that dietary effects tend to overwhelm evidence of this sensitivity.

We also observed several microbiota that stratified exposed and unexposed groups of fish in both diet-robust and diet-dependent manners. Unexposed Gemma-diet fed fish were enriched for *Macellibacteroides* and *Aurantisolimonas* (Table S3.5.2), unexposed Watts-diet fed fish were enriched for an unnamed genus of *Barnesiellaceae, Fluviicola, Paucibacter*, and *Brevibacterium* (Table S3.5.3), and unexposed ZIRC-diet fed fish were enriched for *Macellibacteroides, Bacteroides, Mycobacterium* and unnamed genera of *Barnesiellaceae* and *Sutterelaceae* (Table S3.5.4). Across all the diets, the taxa that were more abundant in unexposed, control fish included *Macellibecateroides, Fluviicola, Bacteroides, Aurantisolimonas, Cerasicoccus*, and three unnamed genera of *Barnesiellaceae, Commonadaceae*, and *Sutterellaceae. Plesiomonas* were more abundant in exposed fish compared to controls (Table S3.5.1). These results indicate that pathogen exposure impacts the abundance of certain taxa within and across the diets. Next, to see if *Mycobacterium* species abundance differed from background, pre-exposure levels we compared *Mycobacterium* abundance between pre-exposure and unexposed control fish to that of exposed fish within each diet. Unexposed Gemma- and ZIRC-diet fed fish had significantly higher abundances of *Mycobacterium* to exposed (Figure 4D, Table S3.5.5). Pre-exposed Watts-diet fed fish had significantly more *Mycobacterium* compared to pre-exposed fish, but they did not differ significantly from unexposed control fish. These results indicate that the abundance of taxa from the genus *Mycobacterium* changes in response to exposure to a pathogenic species in a diet-dependent manner.

## Discussion

Zebrafish are an important emerging model organism for understanding the microbiome. Yet, there is little consistency across studies in terms of the husbandry practices used to conduct zebrafish microbiome experiments, especially in terms of diet. This lack of consistency likely stems from a dearth of knowledge about how different standard zebrafish diets impact study outcomes, both in terms of the gut microbiome’s composition as well as the physiological endpoints of the host. Our study offers critical insight into how three standard zebrafish dietary formulations impacts these outcomes, finding that the zebrafish gut microbiome’s development and response to pathogen exposure is sensitive to diet. These observations help clarify inconsistencies across studies, underscore the importance of considering diet when integrating data across investigations, and inform on efforts to develop standard approaches in zebrafish microbiome research.

We found that diet had a substantial impact on the structure of the gut microbiome in adult zebrafish. Previous research has found that diets with varying compositions of key macronutrients (e.g., protein, lipids and fiber content) impacts zebrafish physiology and the gut microbiome[5, 16–18, 29–32]. Moreover, diet’s effect on restructuring the host’s gut microbiome has been observed across an evolutionarily diverse array of vertebrate and invertebrate animal hosts[8, 9, 11, 12, 33]. However, the nutritional compositions used in these prior studies tend to vary considerably. In particular, the feeds our study considered are far more consistent in their composition than the diets that are typically included in studies of the effect of diet on the gut microbiome (e.g., high-fast v. low-fat diets). Moreover, a unique strength of our study is that fish were fed the same diets over the vast majority of their lifespan (30 to 214 dpf), which is more consistent with a standard husbandry approach that maintains fish on a specific diet than the relatively short-term exposures to different types of diet that are typically employed in related research. Because of these features of our experimental design, our work provides important clarity into how seemingly subtle differences in husbandry practice can result in substantial differences in the composition of the adult zebrafish gut microbiome.

We also found that diet impacts the developmental variation in the gut microbiome. Prior work investigating the successional development of the zebrafish gut microbiome has had inconsistent results; our efforts indicate that these inconsistencies may be attributable to the different diets utilized in these prior studies ^16^·^25^·^26^·^28^. For instance, Stephens *et al*. used a variety of live and dry food diets and found that juvenile zebrafish gut microbiomes were highly diverse but declined with age[30], while Wong *et al*. found opposite results for juvenile zebrafish that were fed defined diets[18]. Furthermore, Burns *et al*. and Xiao *et al*. noted that the observed early life variability of the gut microbiome could be a result of husbandry choices involving diet[29, 31, 32]. While our study differed in exact length and sampling time points as compared to these prior studies, we do find congruent trends in gut microbiome diversity to other zebrafish studies that sampled within similar developmental periods as those interrogated in our investigation. However, it is difficult to directly compare our results to these prior studies because they sampled at different time points, used a variety of diets throughout their study, used diets different from those included in our study, or did not disclose which diets were used. It is worth nothing that while our fish were fed the same diet from 30 days onward, at 114 dpf fish in our study were switched from a juvenile formulation to an adult formulation of their respective diets. These formulations differed slightly in some diets (e.g., Gemma and Watts), but in others more substantially (e.g., ZIRC), which may contribute to the variability we observed in the gut microbiome between diets across zebrafish development. Despite these limitations, we found adult zebrafish fed diets of similar nutritional composition manifest distinct gut microbiome successional patterns in community compositions across adulthood. Future work should seek consistency in diet formulations and increase sampling time points throughout zebrafish development to further clarify the successional development of zebrafish gut microbiomes.

Finally, we observed that the gut microbiome of zebrafish were sensitive to pathogen exposure, but diet was the main driver of gut microbiome structure. We ensured all fish were exposed to the pathogen by injecting *Mycobacterium chelonae* into the coelomic cavities of the fish at 4 mpf. We found that presence of infection was not sufficient to explain associations with microbiome diversity or community composition, which is likely due to being underpowered to detect them. We found infection by diet interactions on a larger number of individuals that were assessed for histopathology, but not with the subset of fish sampled for microbiome analysis. Therefore, having a sufficiently large sample size is important for observing infection effects on the gut microbiome. However, we found that gut microbiome diversification did not change after exposure to *M. chelonae* uniquely in ZIRC-diet fed fish relative to their unexposed controls. Our results contrast our prior work that found exposure to an intestinal helminth was associated with an increase in microbiome diversity[22]. One possible explanation for this discrepancy is our prior study investigated an intestinal helminth which may have different impacts on the gut microbiome associated with differences in intestinal lesion to that of a pathogenic bacterial species. For example, the nematode *Pseudocapillaria tomentosa* penetrates the intestinal epithelium and causes profound pathologic changes[22], whereas disease caused by Mycobacterium species in zebrafish are characterized by extra-intestinal infections[25]. *Mycobacterium* spp in zebrafish are hypothesized to be introduced early in life through ingestion, including diet[28, 34], while fish in our study were exposed by injection into their coelomic cavities at adulthood when their gut microbiomes have been firmly established. Priority effects may have hindered the injected species of *Mycobacterium* from more substantially altering the gut microbiome at adulthood than if it had been introduced through a natural route during early-life microbiome assembly[35]. Future work should consider using a natural mode of infection and exposing fish to a variety of pathogens to elucidate the gut microbiome’s role in mediating pathogen exposure. Furthermore, because we found that the effect of diet was far greater than pathogen exposure on shaping the gut microbiome, future studies must consider diet effects, as they may overwhelm infection effects.

In conclusion, we found diet is one of the most important factors driving variation in the zebrafish gut microbiome. Unlike prior studies, including the extensive research conducted in mammalian models, that have evaluated dietary effects on the gut microbiome using diets that fundamentally differ in macronutrient composition, our work reveals that even relatively consistent diets that are commonly selected as normal husbandry practices elicit these large impacts on microbiome composition. While the zebrafish gut microbiome differs taxonomically from other animal systems, there is a substantial amount of shared functional capacity between zebrafish and mammalian gut microbiomes[36]. Consequently, the taxa-specific associations we found here may not directly translate to other animal systems, but the interactions between the microbiome, diet and pathogen exposure may be similar. Future work should illuminate the underlying mechanisms of the diet’s influence on zebrafish development, gut microbiome structure and the microbiome’s sensitivity to pathogen exposure. Collectively, our study demonstrates that investigators should carefully consider the role of diet in their microbiome-targeted zebrafish investigations, especially when integrating results across studies that vary by diet.

## Conclusions

Collectively, our study demonstrates the effect of commonly used laboratory diets on the gut microbiome of zebrafish. We reared zebrafish across their lifespan on three commonly used diets and analyzed the gut microbiome of juvenile and adult fish. Our findings demonstrate that diet impacts the developmental trajectories of the zebrafish gut microbiome, even with similar nutritional compositions. Additionally, diets were found to sensitize the gut microbiome to pathogen exposure. These results have important implications for the practice of zebrafish husbandry and the selection of diets in microbiome studies. Our findings will also contribute to ongoing discussions about standardizing husbandry practices, including diet, in the zebrafish research community.

## Methods

### Fish Husbandry

A total of 270 30 days post fertilization (dpf) AB line zebrafish were randomly divided into eighteen 2.8 L tanks (15 fish/tank) on a single pass flow-system tanks (15 fish/tank). During the experiment, temperature was recorded daily and ranged from 25.5–28.3°C, with the exception of two isolated overnight temperature drops below that range due to two separate power loss events that affected the source water sump heater. All other water conditions were monitored weekly, pH ranged from 7.0–7.6, total ammonia ranged from 0–0.25 ppm (measured with pH and ammonia API test kits; Mars Fishcare North America Inc. Chalfont, PA), and conductivity ranged from 109 −166 microsiemens. Light in the vivarium was provided for 14 hours/day. One plastic aquatic plant piece approximately 6 inch in length was added to each tank for enrichment when fish were 214 dpf. A stock of similarly aged Casper line fish were maintained for the duration of the experiment, with a third of the stock being maintained on each of the diet regimens matching the AB line zebrafish. These fish served as filler fish and were added to the tanks after each histological sampling time point to maintain the 15 fish/tank ratio required to maintain the prescribed diet volumes per feeding.

### Diets

Fish were all fed the same nursery diet until 30 dpf, a combination of paramecia, brine shrimp, and the ZIRC Nursery Mix: Zeigler AP Larval Diet (Ziegler Bros Inc., Gardners, PA) and freeze dried rotifers. Fish were then transferred to the OSU facility and assigned randomly to one of three juvenile diets: Gemma Micro 150/300 (Skretting, Fontaineles-Vervins, France), Watts High-Fat Juvenile Mix, or ZIRC Juvenile Mix, twice daily (9 AM and 3 PM local time) until 60 dpf. From 60 dpf onward, OSU fish were not fed on weekends and 1-day holidays as per the facility institutional animal care and use protocol. The total quantity fed daily was 3% fish body weight. This continued until fish were 214 dpf and then they were transitioned to the adult version of their previously assigned juvenile diet: Gemma Micro 500 (Skretting, Fontaineles-Vervins, France), Watts Low-Fat Adult Mix, or ZIRC Adult Mix, twice daily (9 AM and 3 PM local time), except weekends and 1-day holidays. The total quantity fed daily was 3% fish body weight. The prescribed amounts of each diet regiment, for both the juvenile and adult diets were delivered by 3D printed spoons specific to the diet and stage of life. These spoons were paired with conical tubes retrofitted with leveling wires to ensure consistent feeding volumes as prescribed. All fish were only fed once, in the afternoons, on sampling days.

### Diet and Pathogen Exposure

Each of the eighteen tanks was assigned one of the three diet regimens: Gemma, Watts, or ZIRC. There were three tank replicates per diet regimens for a total of nine tanks that were exposed to *M chelonae via* intraperitoneal injection (3 tanks/diet with 15 fish/tank). The remaining nine tanks were similarly assigned to diet regimens and were exposed to a sterile 1X-phosphate buffered saline (PBS) solution via intraperitoneal injection. Each fish was injected with 10 uL of either the M *chelonae* inoculum or saline solution. The injections were completed over the course of two days and the *M chelonae* inoculum was prepared as a 0.5 McFarland each day with a target dose/fish of 5 X 10^4^ viable bacteria/fish This target dose was chosen as we have found that it induces a higher prevalence of M *chelonaein* zebrafish with minimal mortality[19, 37, 38].

Day 1 *M chelonae* inoculum was afterwards determined by plating to be 3.1×10^3 dose per fish, while Day 2 *M chelonae* inoculum was determined by plating to be 1.0×10^5 dose per fish. For ZIRC and Gemma, two tanks for ZIRC fish were injected on Day 1, and 1 tank on Day 2. For Watts, one tank was injected on Day 1 (low dose) and 2 tanks were injected on Day 2 (high dose). No significant difference was observed in prevalence was observed so further analyses treated the exposed fish with in each diet group together.

Low and high dose across tanks:

- Gemma:
  - Low: Tank 14 and 35
  - High: Tank 26
- Watts
  - Low: Tank 6
  - High: Tank 12 and 33
- ZIRC
  - Low: Tank 7 and 10
  - High: Tank 4

### Growth Parameters and Sex Determination

Growth and sex parameters were collected when fish were 101–102, 129–130, 213–214 dpf for interfacility comparison. Additionally, these parameters were also collected at 164–165 dpf which was 5 weeks post exposure that were evaluated in comparison to the 213–214 dpf measurements which were 15 weeks post exposure for evaluation of disease effects. Sex was determined by gross differences in morphology and confirmed by histology for all samples collected for disease severity evaluation. Following overnight fecal collection, individual fish would be placed in a pre-anesthetic solution of 50 ppm MS-222 prepared with Tricaine-S (Western Chemical Inc., Ferndale, WA; a subsidiary of Aquatic Life Sciences Inc.) briefly before being transferred to a 150 ppm MS-222 anesthetic solution in a Petri dish on centimeter grid paper to be photographed. Fish were photographed when immobile but still upright. Standard length and width were evaluated via photographs taken with an iPhone (Apple Inc., Cupertino, CA) and analyzed with ImageJ software (https://imagej.net). Weight was obtained while the fish was still under the effects of anesthesia by transferring them from the photography Petri dish to a Petri dish on a scale with a volume of tared fish water, with excess water was removed. Body condition score is a length normalized metric of weight (for equation, see Methods) and serves as a general indicator of health in zebrafish and was calculated using the following equation:

-
$\text{BCS}=\text{Weight }(\text{mg})/\text{Length }{\left(\text{mm}\right)}^{3}\;\times \;100$

### Histopathology

Fish were euthanized by hypothermia preserved in Dietrich’s solution, processed, and slides stained with Kinyoun’s acid-fast[39]. Fish were processed into mid-sagittal sections as previously described[40]. Infection in fish were scored as positive when acid fast bacilli were observed in extra-intestinal organs[40]. A Chi-square test was used to compare positive and negative infections between fish fed each diet.

### Fecal Collection

Five fish from each tank at 4- and 7-months post fertilization sampling time points were randomly selected for fecal sampling. Fecal material was collected from individual fish at the same sample intervals as outlined for the growth parameters. Fecal collection was set up the day before growth parameter sampling. Fish were transferred to 1.4 L tanks (1 fish/tank) containing ~0.4 L of fish water at least 30 minutes after the last feeding of the day. Fish were left to defecate overnight and all fecal material was collected from each tank the following morning in a 1.5ml microcentrifuge tube. Fecal samples were immediately spun at 10k rpm for 2 minutes, excess tank water was removed, and samples were snap frozen on dry ice and stored at −80 °C until processing.

### 16S Sequencing

Microbial DNA was extracted from zebrafish fecal samples and 16S rRNA gene sequence libraries were produced and analyzed following established approaches[41]. Briefly, the DNeasy PowerSoil Pro DNA kits (Qiagen) were used to extract and purify DNA. The V4 region of the 16S rRNA gene was PCR amplified using the Earth Microbiome Project 16S index primers and protocols (Walters et al., 2016). PCR products were visualized on a 1.5% agarose gel and quantified on a Qubit 2.0 (Thermofisher Scientific) using the Qubit dsDNA HS Assay. One hundred nanograms of PCR product for each DNA sample was pooled and cleaned using the QIAquick PCR Purification Kit (Qiagen). The quality of the pooled library was verified on the Agilent TapeStation 4200. The prepared library was submitted to the Oregon State University Center for Quantitative Life Sciences (CQLS) for 300 bp paired-end sequencing on an Illumina MiSeq System (RRID:SCR_016379).

### Statistical Analysis

All microbiome DNA sequence analyses and visualizations were conducted in R (v 4.2.1)[42]. Fastq files were processed in using the DADA2 R package (v 1.18.0)[43]. Briefly, forward and reverse reads were trimmed at 250 and 225 bp, respectively, subsequently merged into contigs, and subject to amplicon sequence variant (ASV) identification. ASVs unannotated at the Phylum level were removed to result in 2029 remaining detected ASVs. We used Wilcoxon Signed-Ranks Tests to identify parameters that best explained the variation in weight and body condition scores. Alpha-diversity was calculated using the estimate_richness function (Phyloseq v 1.38.0) and transformed using Tukey’s Ladder of Powers using methods described previously[41]. After transformation, scores were normalized from 0 to 1 by dividing each score by the maximum value, which allowed us to compare results across alpha-diversity metrics using general linear models (GLMs). Post-hoc Tukey Tests evaluated pairwise comparisons of models using multcomp (v1.4–2) glht function[44]. We corrected for multiple tests using Benjamini-Hochberg correction[45]. Two-way ANOVA assess these GLMs. Beta-diversity models were generated using methods described previously[41]. Briefly, we evaluated three beta-diversity metrics–Bray-Curtis, Canberra, and Sørensen and resolved the relationship between experimental parameters and beta-diversity by applying a step-wise model selection approach as implemented in the capscale function (vegan package v 2.5)[46]. Optimal models were subsequently subject to PERMANOVA analysis to determine if the selected model parameters significantly explained the variation in microbiome composition across samples. Differential abundance was measured using ANCOM-BC (v 2.0.1)[47].

## Data Availability

All code generated during this analysis is available in the ZF Diet Infection 2020 repository at the following URL: https://github.com/sielerjm/ZF-Diet_Infection. The raw sequence files generated during the current study are available at the NCBI Sequence Read Archive (SRA) project numbers PRJNA929305.

## Abbreviations

dpf: days post fertilization
mpf: months post fertilization
ZIRC: Zebrafish International Resource Center
